# Supplementary material for: Artificial intelligence development races in heterogeneous settings
Source: Sci Rep. 2022 Feb 2;12:1723. doi: 10.1038/s41598-022-05729-3 (PMC8810789; doi:10.1038/s41598-022-05729-3)
Supplement: Supplementary file 1 — Supplementary Information. [file 41598_2022_5729_MOESM1_ESM.pdf]

Supplementary Information: Artificial Intelligence Development Races in Heterogeneous Settings

Theodor Cimpeanu[1], Francisco C. Santos[3], Luís Moniz Pereira[2], Tom Lenaerts[4,5,6,7], and The Anh Han[1,⋆]

[1] School of Computing, Engineering and Digital Technologies, Teesside University, Middlesbrough, UK TS1 3BA

[2] NOVA Laboratory for Computer Science and Informatics (NOVA-LINCS), Faculdade de Ciências e Tecnologia, Universidade Nova de Lisboa, 2829-516 Caparica, Portugal

[3]INESC-ID and Instituto Superior Técnico, Universidade de Lisboa, Portugal

[4] Machine Learning Group, Université Libre de Bruxelles, 1050 Brussels, Belgium

[5] Artificial Intelligence Lab, Vrije Universiteit Brussel, 1050 Brussels, Belgium

[6] Center for Human-Compatible AI, UC Berkeley, Berkeley, 94702, USA

[7] FARI Institute, Université Libre de Bruxelles-Vrije Universiteit Brussel, 1050 Brussels, Belgium

⋆ Corresponding author: The Anh Han (T.Han@tees.ac.uk)

Additional simulation results

To further illustrate the key differences between each type of network, we plot typical simulation runs for different p_r risk probability values in the area (**II**) of the early AI race (see Figure S1). It is immediately apparent that the two un-normalised scale-free networks provide significant improvements in safety compliance in the dilemma zone. This is further compounded by the effect of clustering on the threshold at which safe development becomes evolutionarily stable. Specifically, we note that when the risk of a disaster occurring due to inadequate safety compliance is intermediate (see, e.g. $p_r = 0.5$ and 0.65), we see a definitive improvement in highly clustered networks (i.e. DMS) as opposed to the basic BA model.

Figure S2 confirms the similar trends encountered in the regular square lattice. There are some very minor differences, but there is very little difference between well-mixed, the normal four-neighbour lattice and the eight-neighbour lattice. We confirm the similar late convergence found previously in some cases of the regular lattice.

We see very few improvements over the previously mentioned results on homogeneous populations. Interestingly, there is an area in the late regime where this type of normalised scale-free network produces more unsafe results (undesirably so) than either the well-mixed or lattice variants. We see some slight improvements in area **(II)** of the early regime.

In order to better understand the role and influence of highly connected zealots in the population, as well as to explore any potential for a government or regulatory agency to interfere in the AI race, we artificially accelerate or fund the safety zealots that had been introduced previously. For this analysis, we choose a small number (10% of high-degree nodes) of individuals, to check whether a very small minority can be exploited by an external investor. In addition to the introduction of players following pathological safe behaviour, we either accelerate their development (similarly to how unsafe players gain increased speed, in this case we add $\frac{sB}{W}$ to the influential pathological players' payoffs, where $s = 2$), or heavily invest in these players (to the extent that other players will always imitate them, by increasing their payoffs by a very large amount 10^7). Figure S7 displays our findings - with very slight improvement throughout. Each approach has its merits in different regions of the early regime, and we see the effectiveness of funding highly connected nodes when the risk for disaster is low. On the other hand, a high risk improves the efficacy of speeding up the development for these dedicated minorities. We note that targeting a very small minority of highly influential players is not sufficient to mitigate the race tensions entirely. Further exploration on this topic would provide more insight into how

external interference can be deployed efficiently.

We study a comprehensive view of pathological players (zealots) planted in a well-mixed network (see Figure S4), but in this case modifying 10% of the total population (not just highly connected nodes). We remove the pathological players from the frequency average to show how these affect the remainder of the population. We see very little effect of pathological players and we suggest that much lower β values would be required to see an effect. With the addition of mutation and more stochasticity, it would be possible for these pathological players to have a significant impact on the outcome.

Figure S5 shows the evolution over time of unsafe behaviour (AU) in the dilemma zone of an early AI race for different environments (corresponding to varying probability values of a disaster caused by insufficient safety regulation, p_r). High-degree individuals appear to have a higher tendency towards safety compliance (at equilibrium) when compared to their lowly or moderately connected counterparts, except for region (**III**), where highly connected individuals are driving to innovate (optimally so). In spite of this, we see the same trends for regions (**I**) and (**III**). However, in region (**II**), highly connected individuals become important leaders in the shift from unsafe to safe behaviour in the AI race. Specifically, for large p_r values (see $p_r = 0.65$; $p_r = 0.78$), there is an evident disparity between the high degree individuals and the bulk of the population, and indeed, this is the region in which heterogeneity improves safety compliance the most. For low p_r values, heterogeneity fails to improve the outcome, but it does serve as an equaliser for intermediate risk values ($p_r = 0.5$). Regulatory actions would therefore still be required to constrain developers when heterogeneity cannot improve safety enough in region **II**, in the case of low risk of disaster to occur.

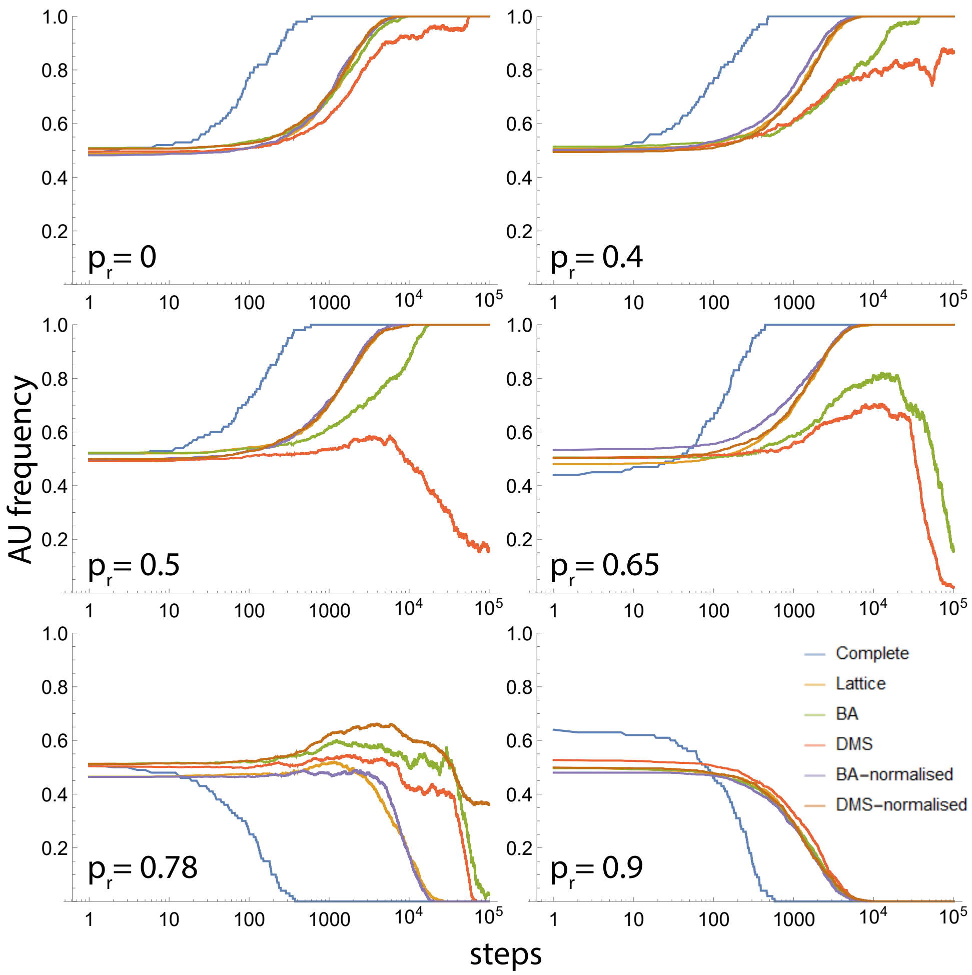

Figure S1. Scale-free networks (especially highly clustered networks) reduce unsafe behaviour in the dilemma regions of the early race, shown using typical runs for different risk probability values, for each type of network. Parameters: $c = 1,\ b = 4,\ B = 10^4,\ \beta = 1$.

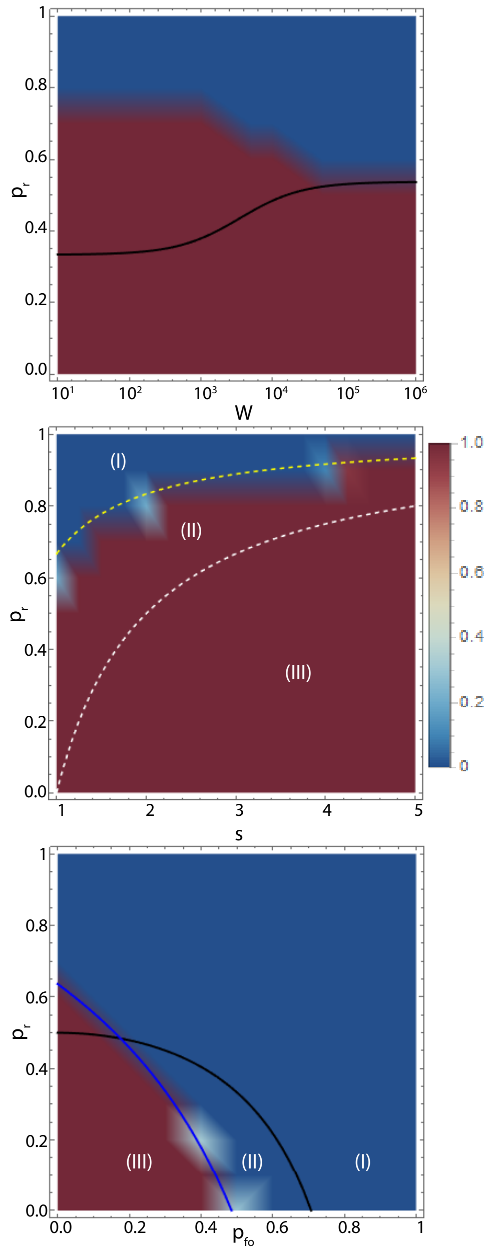

Figure S2. Total AU frequencies for the 8-neighbours lattice. The top row reports the spectrum between an early and late AI race (for varying W, with $p_{fo} = 0.1,\ s = 1.5$), the middle row addresses the early regime for varying s and p_r ($p_{fo} = 0.5,\ W = 100$), and the bottom row addressees the late regime for varying p_{fo} and p_r ($s = 1.5, W = 10^6$). Other parameters: $c = 1,\ b = 4,\ B = 10^4,\ \beta = 1$.

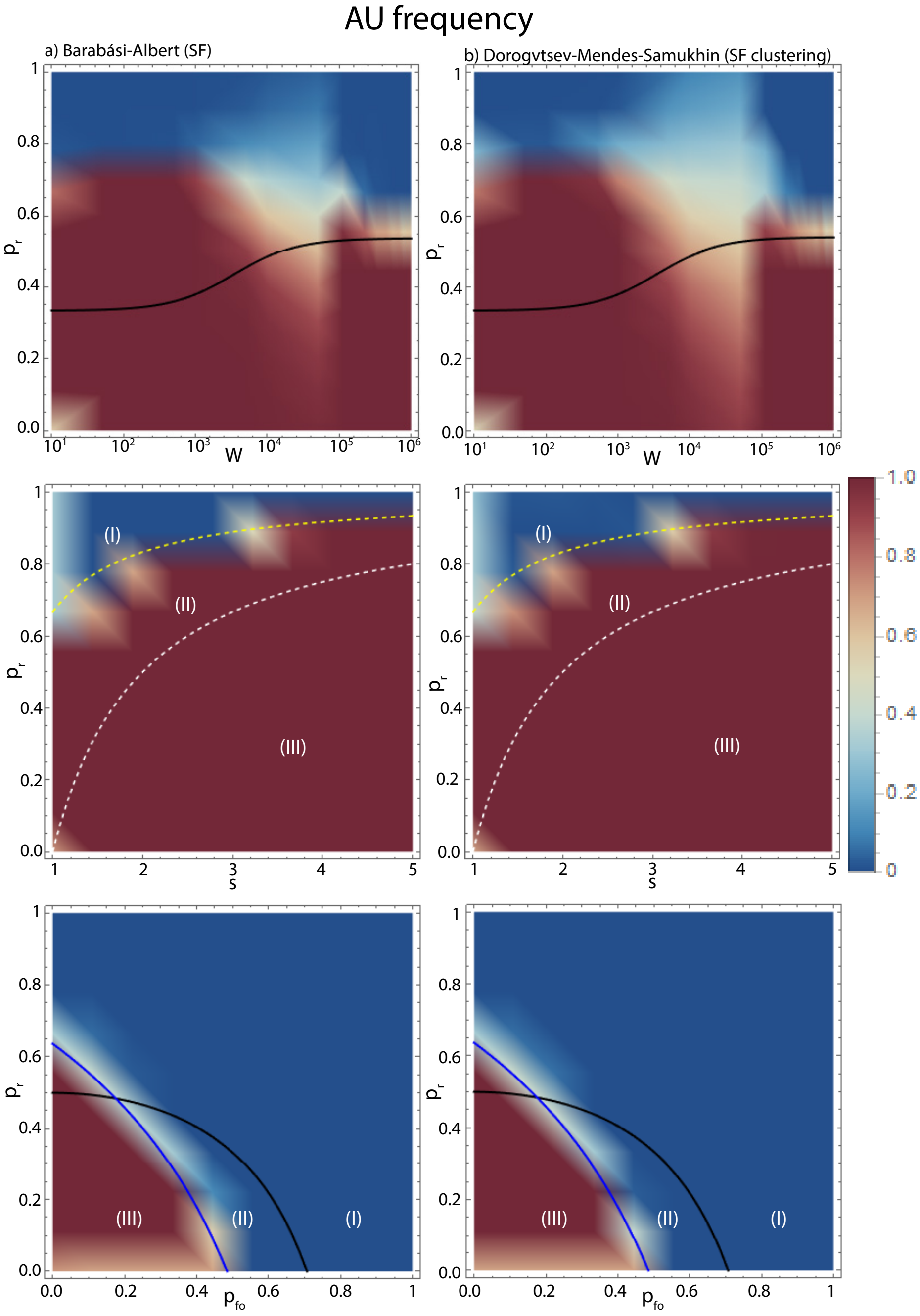

Figure S3. Comparison between the two different scale-free networks, BA and DMS. In this case, the payoffs have been normalised. The top row reports the spectrum between an early and a late AI race ($p_{fo} = 0.1,\ s = 1.5$), the middle row addresses the early regime in more detail ($p_{fo} = 0.5,\ W = 100$) and the bottom row considers a late AI race ($W = 10^6,\ s = 1.5$). Parameters: $c = 1,\ b = 4,\ B = 10^4,\ \beta = 1$.

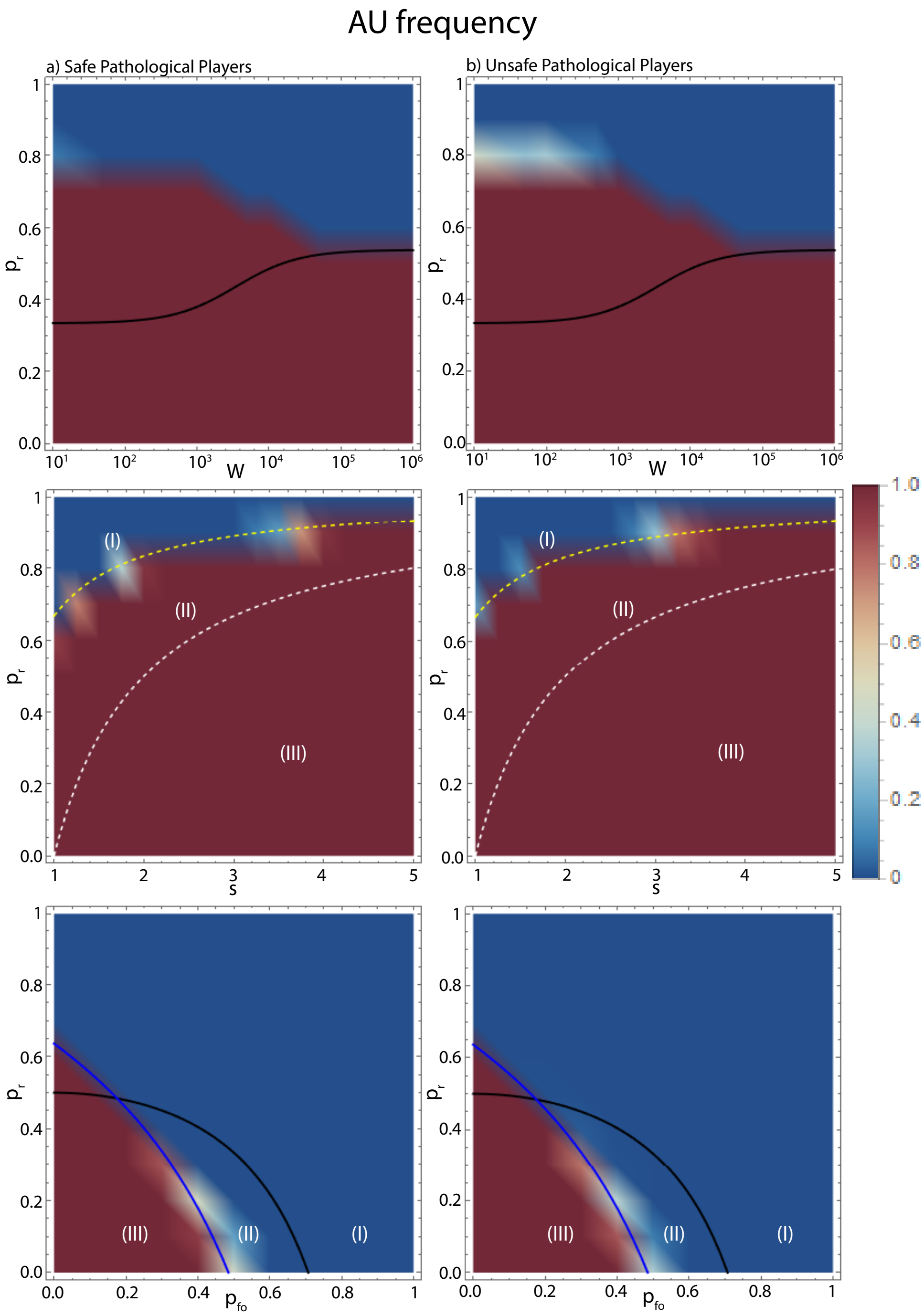

Figure S4. Introducing safe and unsafe zealots in the well-mixed scenario. Please note that the pathological players are excluded from these frequencies. The top row reports the spectrum between an early and a late AI race ($p_{fo} = 0.1,\ s = 1.5$), the middle row addresses the early regime in more detail ($p_{fo} = 0.5,\ W = 100$) and the bottom row considers a late AI race ($W = 10^6,\ s = 1.5$). Parameters: $c = 1,\ b = 4,\ B = 10^4,\ \beta = 1$.

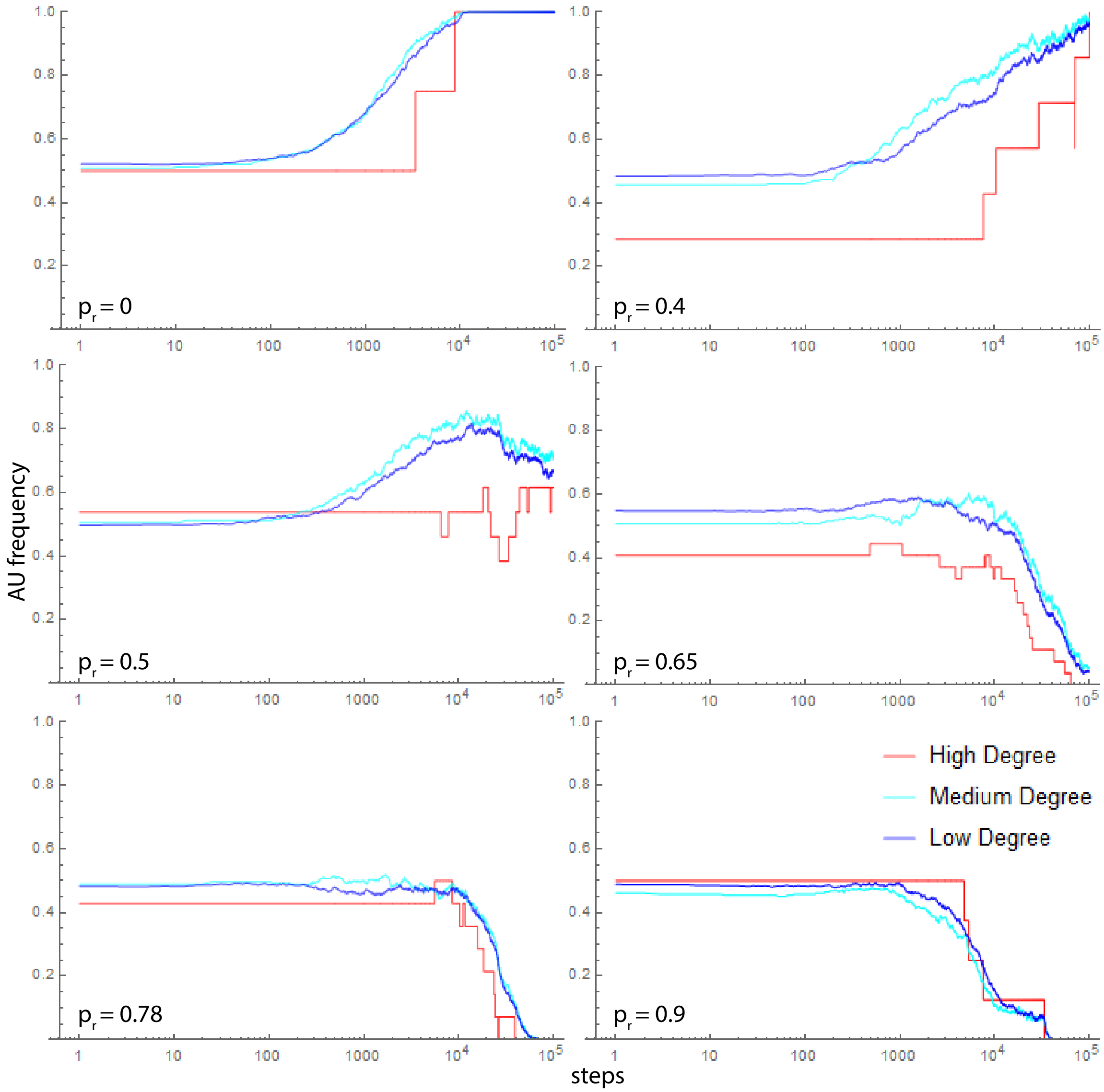

Figure S5. Typical runs showing the distribution of unsafe behaviour (AU) in an early AI race, grouped by degree class (connectivity) of the nodes on DMS networks, for different risk probabilities. Parameters: $c = 1,\ b = 4,\ s = 1.5,\ B = 10^4,\ W = 100,\ \beta = 1.$

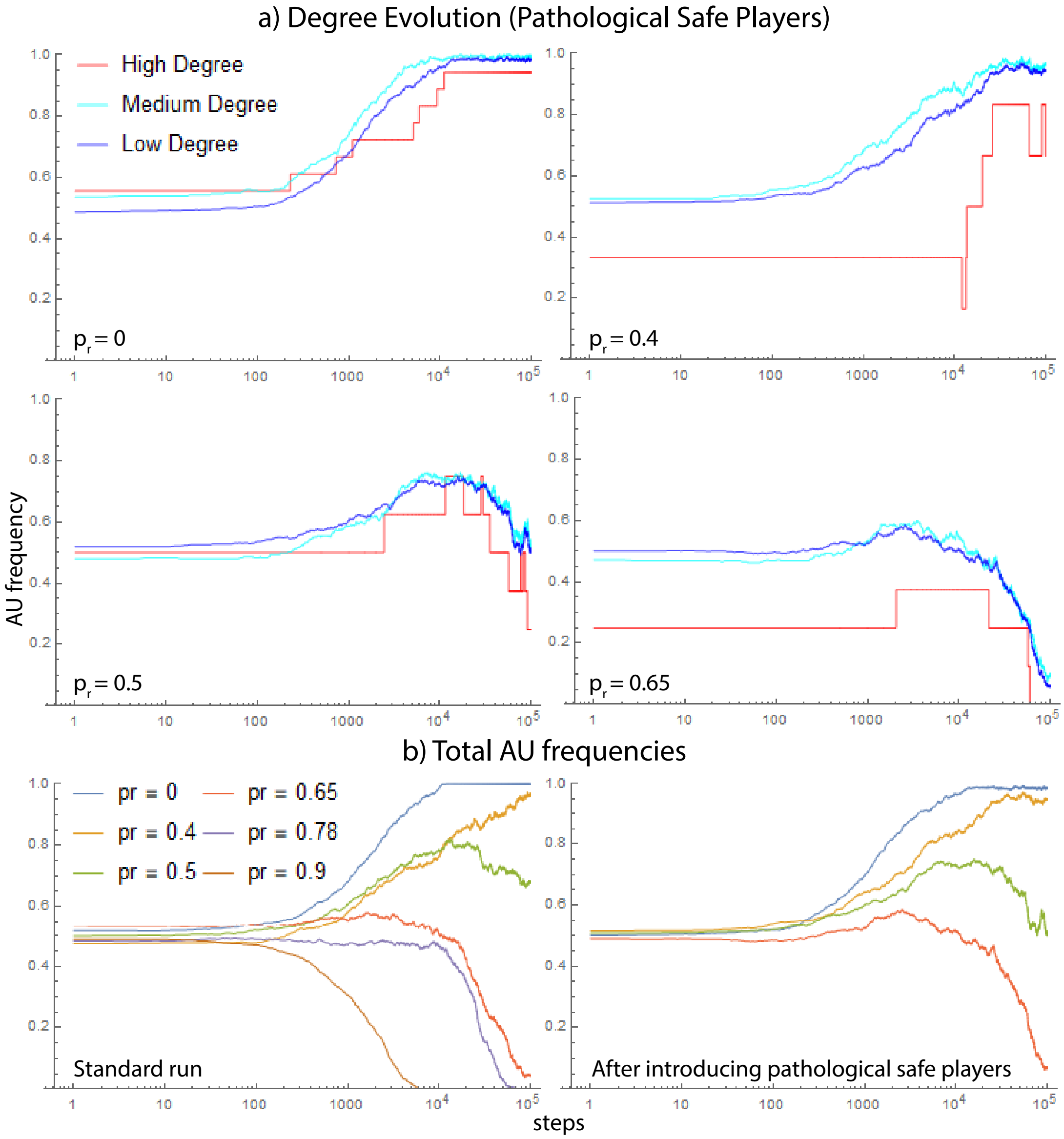

Figure S6. Typical runs exploring the evolutionary degree distribution of unsafe behaviour in an early AI race, following the introduction of safety zealots (pathological safe players) in the population of DMS networks. We randomly allocate 10% of high degree individuals as safety zealots. Note that we measure the frequency for the whole population, including the pathological players. Parameters: $c = 1,\ b = 4,\ s = 1.5,\ B = 10^4,\ W = 100,\ \beta = 1$.

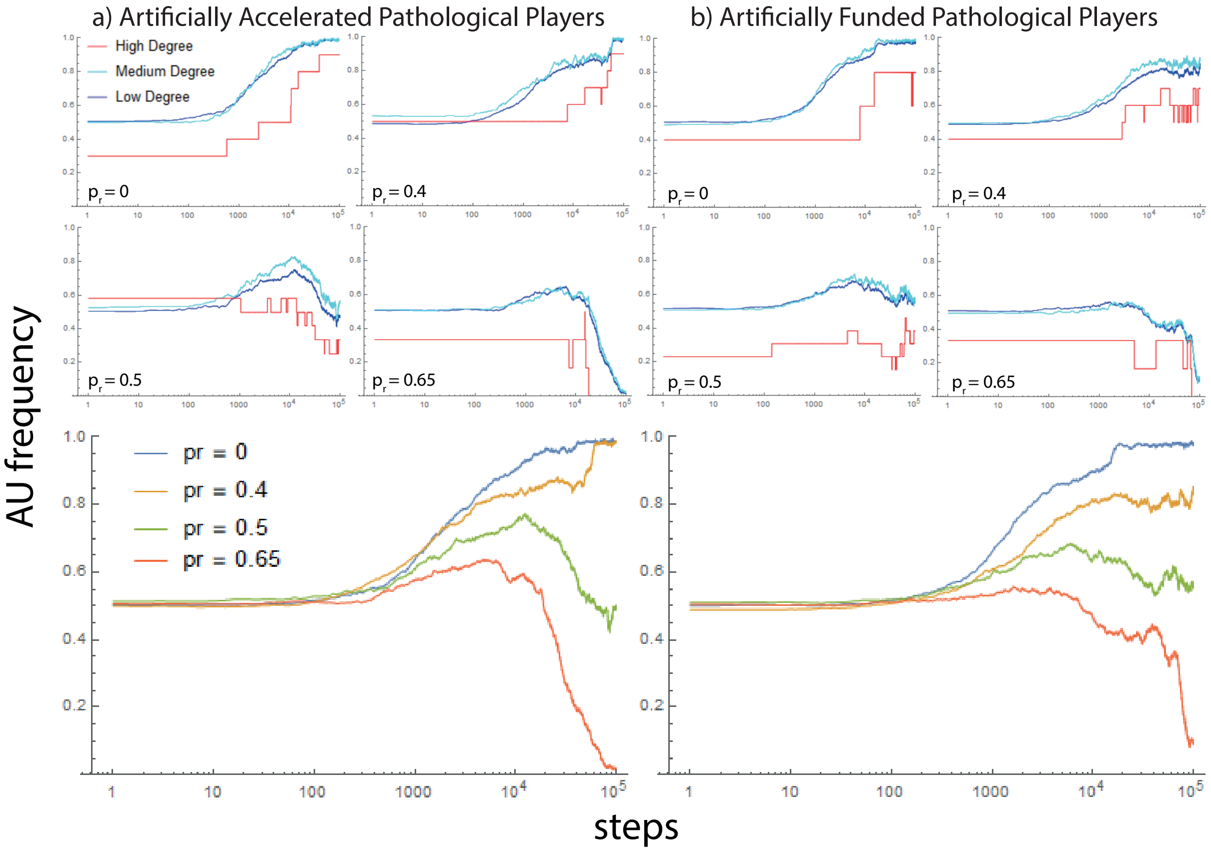

Figure S7. Typical runs exploring the evolutionary degree distribution of unsafe behaviour in an early AI race, following the artificial acceleration (or funding) of safety zealots (pathological safe players) in the population interacting in DMS networks. We randomly allocate 10% of high degree individuals as safety zealots. Note that we measure the frequency for the whole population, including the pathological players. Parameters: $c = 1,\ b = 4,\ s = 1.5,\ B = 10^4,\ W = 100,\ \beta = 1.$

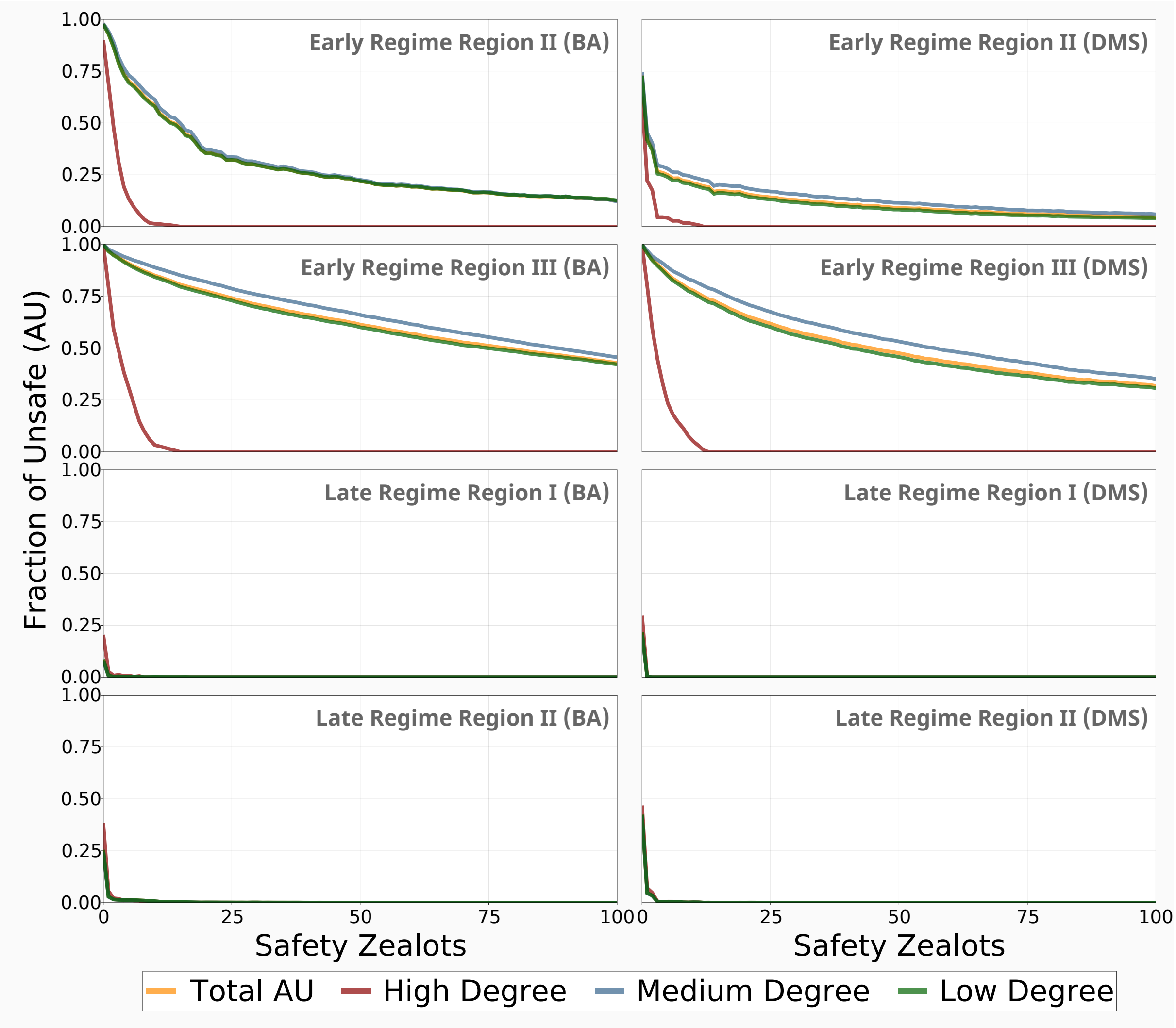

Figure S8. Hubs prefer slower, thus safer developments in the early race, and this can be further exploited by progressively introducing safety zealots in highly connected nodes. We show the results for both regimes, as well as the appropriate regions where safety (early region II and late region I), and conversely where innovation (early region III and late region II) are the preferred collective outcomes. The top four panels report the results for the early regime ($p_{fo} = 0.5,\ W = 100$ with $p_r = 0.5$ for region II and $p_r = 0.1$ for region III), and the bottom four do so for the late regime ($p_{fo} = 0.6,\ W = 10^6$ with $p_r = 0.3$ for region I and $p_r = 0.1$ and region II). Other parameters: $c = 1,\ b = 4,\ B = 10^4,\ s = 1.5,\ \beta = 1.$

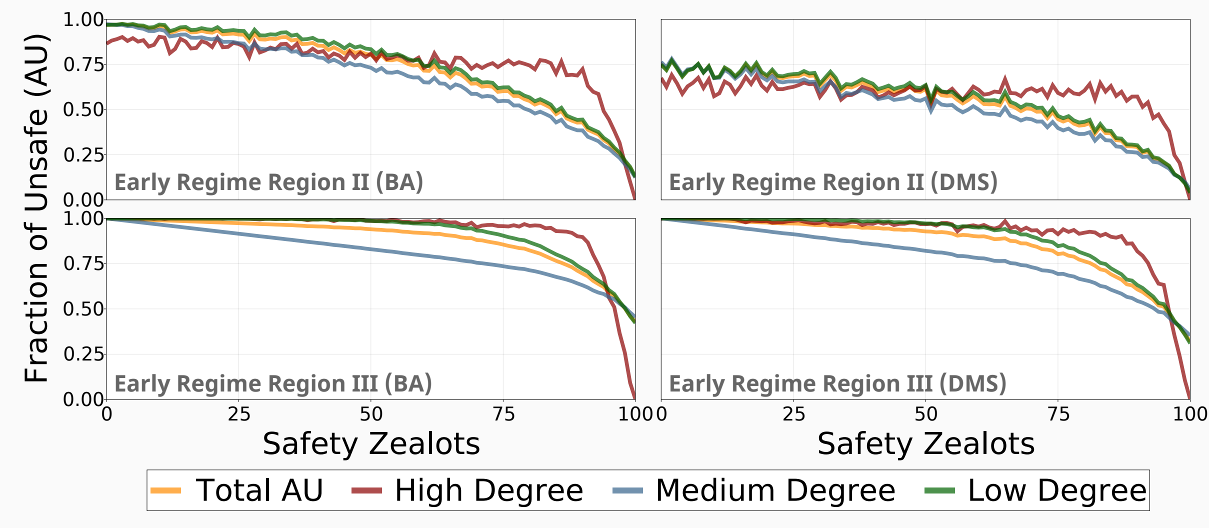

Figure S9. Introducing safety zealots in reverse order (still selecting the top 10% of nodes based on degree connectivity) does not produce the same exponential increase in safety that we had seen in Figure 3. We show the results for the early regime, as well as the appropriate regions where safety (region II), and conversely where innovation (region III) are the preferred collective outcomes. Parameters are $p_r = 0.5$ for region II and $p_r = 0.1$ for region III, chosen for clear presentation. Other parameters:
$c = 1,\ b = 4,\ B = 10^4,\ s = 1.5,\ \beta = 1,\ p_{fo} = 0.5,\ W = 100.$
